# Induction Murine Models of Chronic Fatigue Syndrome by *Brucella abortus* Antigen Injections: Is Anemia Induced or Not?

**DOI:** 10.1155/2015/191489

**Published:** 2015-06-11

**Authors:** Junji Moriya, Qiang He, Hiroaki Uenishi, Sumiyo Akazawa, Jun-ichi Yamakawa, Junji Kobayashi, Yasuhito Ishigaki

**Affiliations:** ^1^Department of General Internal Medicine, Kanazawa Medical University, Ishikawa 920-0293, Japan; ^2^Division of Molecular and Cell Biology, Kanazawa Medical University, Ishikawa 920-0293, Japan

## Abstract

To investigate whether* Brucella abortus* (BA) antigen injections lead to anemia, and to establish an appropriate Chronic Fatigue Syndrome (CFS) animal model by BA injections, 6 repeated injections of BA antigen were fulfilled every 2 weeks. At a high dose of 1*∗*10^10^ particles/mouse, anemia was induced within 2 weeks and then recovered a lot at the end of the research, while at a moderate dose of 1*∗*10^8^ (3 injections) shifting to 1*∗*10^9^/mouse (3 injections) anemia was absent. In both groups running wheel activity remained very low even 6 weeks after the last injection.

## 1. Introduction

CFS is a complex illness defined by unexplained disabling fatigue and a combination of nonspecific accompanying symptoms, such as unusual postexertional malaise, nonrefreshing sleep, significant impairment in memory/concentration, headache, muscle pain, joint pain, sore throat, and tender lymph nodes, most of which are neural and psychiatric symptoms [1, 2]. Although there is a growing body of research directed toward understanding the biology of fatigue, the etiology is still unclear. It is generally believed that multiple systems are involved in CFS (central nervous system, endocrine system, and immune system), among which the immune system was always emphasized. This is supported by the observation that the onset of CFS is often preceded by infections and a “flu-like” illness. An alteration in cytokine profile, a decreased function of natural killer (NK) cells, and a reduced response of T cells to mitogens and other specific antigens [3–7] have been reported.

Building animal models of CFS helps researchers to better understand the pathogenesis and to test many new therapies. An ideal animal model of fatigue should encompass as many attributes of the human experience of fatigue as possible. Unfortunately, until now no perfect animal models exist. The researcher could emphasize one aspect of human CFS, like an immune stimulus (viral infection), environmental stress (heat exposure), or physical fatigue (forced exercise). Most studies used the immunologically induced fatigue [8].

In 1998 Ottenweller and his colleagues first developed a CFS animal model by treating the mice with killed* Brucella abortus* (BA) antigen [9]. After the injection of 8*∗*10^8^ particles of BA, the mice running wheel activity was decreased and recovered 3 weeks later. We improved this method by more BA injections (6 times) to prolong the course of fatigue that mimics the real chronic course of human CFS [10, 11]. At a 1*∗*10^10^ density, BA was injected every two weeks. After the last injection the suppression of running wheel activity lasted at least 7 weeks.

On the other hand, several recent reports indicated that mice injected with BA antigen (5*∗*10^8^/mouse, single) may develop anemia over 10 days that recovers in 3–6 weeks [12–14]. This prompted us to determine whether BA injections will cause anemia which may suppress the running activity. The objective of this research was to use BA to induce fatigue as measured by decreased wheel running without evidence of anemia.

## 2. Materials and Methods

### 2.1. Animals and Living Conditions

Eight-week old BALB/c mice (female, 20–24 g, CLEA Japan, Tokyo) were housed 6–8 per home cage on reversed 12 h light : 12 h dark cycles (lights off at 19:00). Ambient temperature was maintained at 22-23°C. Food and water were available ad libitum.

Running wheel activity was observed in a special cage including a running wheel (23 cm in diameter), counters, cribs, and water taps (detailed description of this cage was in our prior research [10]). The mice were free to stay in the rest area or enter the running wheel. 32 mice were divided into High density group (H group, *n* = 12), Minor density group (M group, *n* = 12), and Control group (C group, *n* = 8). Each of them was put into the running wheel cage singly for 24 h at least 3 times (at least 3 days interval) to reduce error variance in the running wheel activities. This adaption period lasted for 2 weeks. Our research protocol was approved by the Animal Experimental Committee of Kanazawa Medical University.

### 2.2. Inductions of CFS Model

Fixed killed whole* B. abortus* ring test antigen was obtained from the National Veterinary Services Laboratories in the United States Department of Agriculture. CFS was induced by six repeated injections of BA antigen solution (0.2 mL per mouse) intraperitoneally every 2 weeks. The stock solution was resuspended in saline so the appropriate dose of killed bacteria could be made. The dose for the H group was 1*∗*10^10^ particles/mouse, while the first 3 injections for M group were 1*∗*10^8^ particles, and the following 3 injections were 1*∗*10^9^. The C group was injected with 0.2 mL saline vehicle 6 times.

Mice were put into the running wheel cages the day before and one day after each injection for 24 h (in and out at 13:00). Then they were taken back to the home cages where they lived gregariously.

Blood Hb was measured of every mouse in each group at 0, 2, 8, 12, and 16 weeks. Tail vein puncture was adopted to minimize the harm to the mice and each time about 30 *μ*L blood was taken to fulfill the test. Mice were killed at the end of 16 weeks (6 weeks after the last injection).

### 2.3. Statistical Analysis

SPSS was used to analyze the data. Independent Student's *t*-tests and ANOVA were performed between the H, M, and C groups. *p* < 0.05 was set to be significant.

## 3. Results

**Figure 1:**
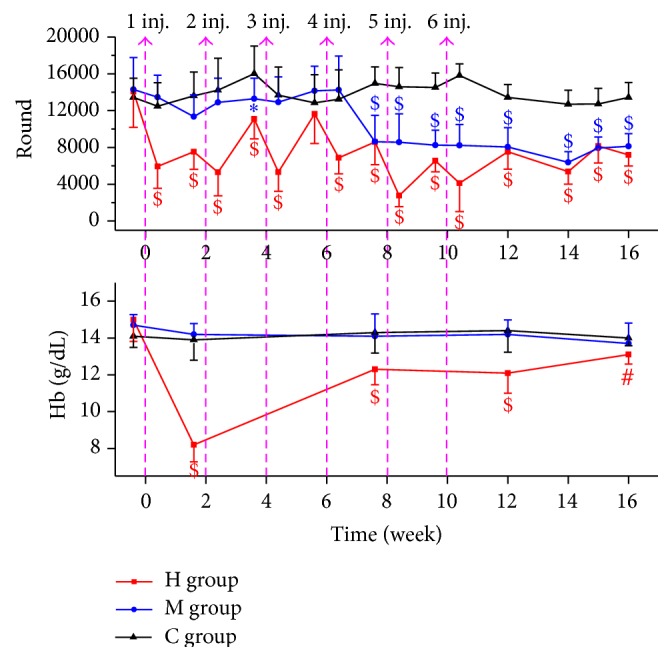
Running wheel activities and Hb levels during time (compare to C group ^*∗*^ *p* < 0.05, ^#^ *p* < 0.01, and ^$^ *p* < 0.001).

At the baseline the running wheel activities (H : M : C, 14085 ± 3908 : 14291 ± 3473 : 13430 ± 2102, *F* = 0.162, *p* = 0.851) and the Hb (g/dL; H : M : C, 15.0 ± 1.19 : 14.7 ± 0.58 : 14.1 ± 0.60, *F* = 2.368, *p* = 0.111) did not differ between each group (Figure 1). During the injections 2 mice died in the H group (after the 3rd and 6th injection), and 1 died in the M group (after the 4th injection).

According to the H group, the first injection was the most potent. It caused an obvious decrease of running activity and Hb level (to 42.1% and 54.7% according to baseline). Although there was a second and third injection, the running wheel activity recovered significantly at 4 (78.7%) and 6 weeks (82.6%). The fourth injection was a hinge, after which the running activity never recovered to over 60% till the end. The worst running performance which was observed after the 5th injection remained only 19.6% according to baseline, while at that point the Hb level had already recovered to over 80%. At the end point the Hb level recovered to 13.1 ± 0.52, which was similar to the C group (14.0 ± 0.42) although the difference still remained significant (*p* = 0.0013), while the running wheel activity still remained low (51.1%).

The first 3 injections (1*∗*10^8^) to the M group contributed nearly nothing to decrease the activities, and the minimum running wheel number was only 79.4% of baseline. Again the 4th injection (dose increased to 1*∗*10^9^/mouse) was the hinge. But the pattern was different: after the 4th injection the activity was not decreased on the second day. 2 weeks after the 4th injection it was reduced to 60.4%, and it never went up over this level. After the 4th injection the running wheel activities kept decreasing for 6 weeks, till the lowest point at 14 weeks (44.7% of baseline), and recovered slightly at the end (56.9%). There was no reduction in Hb level during all the 6 injections in the M group compared with the C group.

## 4. Discussion

As far as we know, this is the first research considering the Hb level during the model building work with BA injection. It was believed that BA injections disturbed the immune system and induced fatigue, while anemia was ignored [9, 11, 15]. According to our results, 1*∗*10^10^ particles/mouse induced severe anemia within 2 weeks, while 1*∗*10^8^/mouse induced no anemia at all. In our pilot study we also tried an even lower density (4*∗*10^7^/mouse); 2 weeks later there was not any decrease in Hb level (*n* = 12, data not shown). In addition it was also found by Sasu et al. that a density of 5*∗*10^8^/mouse decreased Hb level remarkably [12]. Based on these findings we arbitrarily believe a higher concentration of BA is necessary to generate anemia. Most probably 5*∗*10^8^ is the critical value. Regrettably in the past most of the research adopted higher concentrations (4*∗*10^8^–1*∗*10^10^/mouse) which make it hard to distinguish which suppressed the running activities, the immune disturbance, the anemia, or both [9, 11, 16, 17].

In 2010 Sasu et al. first described 5*∗*10^8^ particles/mouse induced anemia, and the Hb level was lowest about 14 days after injection [12]. Later in 2014 Gardenghi et al. [13] and Kim et al. [14] reported similar results. The anemia will not recover until 3–6 weeks thereafter according to their research. In the H group, the anemia also began to improve at 8 and 12 weeks, even though there were more injections. However, full recovery will not be observed until 2 weeks after the last injection. This indicated that the anemia seemed to be more like an acute reaction to the BA injection, and there were little cumulated effects. Decrease in the running wheel activities displayed a different pattern. The running wheel activities displayed both acute and cumulated effects during the injections. At 8 weeks (after the 5th injection) the running activity remained only 19.6% of baseline. The variations of Hb level and running wheel activity were similar after first injection, then different during the following injections. One possible explanation is that anemia may suppress the activity at the beginning, while as time passed by, this suppression effects shift from anemia as the cause to other factors, most probably the immune disturbance. However 2 mice died during the 6 injections and even more died in our previous research [10, 11]. It is apparent that this density (1*∗*10^10^ particles/mouse) was too high, which resulted in anemia and was deadly for mice.

This prompted us to improve the animal model building work. Lower concentrations were preferred. Based on other research and our pilot study we made a decision that starting at a dose of 1*∗*10^8^ particles/mouse and then increasing to 1*∗*10^9^ may be a suitable choice. In the M group anemia was absent from beginning to the end, while running wheel activity decreased to about 60% from 12 weeks until the end, which was very similar to the H group. One main difference between the H and M groups is that there was an obvious decline in running activity after each injection in the H group, which was absent in the M group. There was only one obvious decline in the M group: after the 4th injection, and the decline appeared chronically instead of as the acute reaction seen in the H group. Compared to H group, the M group is a more successful method as there was no anemia at the beginning and it mimics the human CFS better, which always begins with a subacute or chronic course.

While single injection of antigen was adopted in most of the other research, it was more like an “acute immunological reaction” instead of chronic fatigue and will recover soon. We believe the pathological changes in our M group model will mimic the human CFS better. We are planning to check the pathogenesis in the nervous and immune system and test some drugs with this animal model.

The results of our study are subject to a number of limitations. First, the Hb level was checked on only 5 time points, which leads to a rough Hb value line during research. The slight variation in Hb was ignored. Second, in our research BA antigen was injected intraperitoneally, using the same method as Sasu et al. [12–14], but different from that used by Ottenweller et al. [9]. Tail vein injection was employed in his research. We failed to check the differences between the two methods. Third, BALB/c mice were adopted in our research, the same mouse strain that Ottenweller used, but C57BL/6 was used in the research by Sasu et al. We are not certain if the results were affected because of mouse strain difference.

Future research should focus on improving the animal model of CFS. For example, combination of different antigens (polycytidylic acid (poly I: C), lipopolysaccaride (LPS), and other bacteria) and even environment stress should be considered.
